# Supplementary material for: The impact of tocilizumab on respiratory support states transition and clinical outcomes in COVID-19 patients. A Markov model multi-state study
Source: PLoS One. 2021 Aug 12;16(8):e0251378. doi: 10.1371/journal.pone.0251378 (PMC8360516; doi:10.1371/journal.pone.0251378)
Supplement: S1 Table — (DOCX) [file pone.0251378.s001.docx]

**S1 Table. Adjusted effect of other covariates on change of state risks**

| **Change of state ^1^** | **Age - 5 years increment** | | **Gender**  **M vs F** | | **SOFA - 1 point**  **increment** | | **Time from**  **COVID19 onset**  **- 1 week increment** | | **Glucocorticoids**  **Yes vs No** | | **Lopinavir / Darunavir**  **Yes vs No** | |
| --- | --- | --- | --- | --- | --- | --- | --- | --- | --- | --- | --- | --- |
|  | **HR** | **95% CI** | **HR** | **95% CI** | **HR** | **95% CI** | **HR** | **95% CI** | **HR** | **95% CI** | **HR** | **95% CI** |
| From NRS to OT | 1.03 | 0.98 - 1.09 | 0.71 # | 0.52 - 0.98 | 1.36 ## | 1.29 - 1.43 | 0.95 | 0.83 - 1.08 | - | - | - | - |
| From NRS to Recovered | 0.80 ## | 0.69 - 0.91 | 0.31 ## | 0.13 - 0.74 | 0.55 # | 0.30 - 0.99 | 0.72 | 0.44 - 1.18 | - | - | - | - |
| From OT to NIV / IMV | 0.93 | 0.85 - 1.02 | 1.36 | 0.78 - 2.38 | 1.31 ## | 1.21 - 1.41 | 1.08 | 0.86 - 1.35 | 0.73 | 0.38 - 1.40 | 1.39 | 0.75 - 2.57 |
| From OT to NRS in recovery | 0.84 ## | 0.77 - 0.91 | 1.10 | 0.67 - 1.80 | 0.65 ## | 0.53 - 0.80 | 0.91 | 0.73 - 1.14 | 0.77 | 0.38 - 1.57 | 1.25 | 0.71 - 2.21 |
| From OT to Recovered | 0.90 | 0.78 - 1.03 | 0.73 | 0.35 - 1.53 | 0.92 | 0.72 - 1.19 | 0.99 | 0.66 - 1.49 | 0.71 | 0.24 - 2.12 | 1.43 | 0.53 - 3.86 |
| From OT to Death | 1.47 ## | 1.11 - 1.95 | 3.62 | 0.75 - 17.41 | 1.13 | 0.88 - 1.45 | 0.99 | 0.59 - 1.66 | 4.52 # | 1.20 - 16.92 | 2.09 | 0.46 - 9.60 |
| From NIV / IMV to OT in recovery | 0.85 ## | 0.75 - 0.96 | 0.63 | 0.33 - 1.22 | 0.88 | 0.75 - 1.03 | 1.19 | 0.85 - 1.68 | 1.53 | 0.82 - 2.84 | 1.61 | 0.78 - 3.33 |
| From NIV / IMV to Death | 1.50 ## | 1.20 - 1.88 | 1.22 | 0.41 - 3.67 | 1.31 ## | 1.10 - 1.55 | 0.81 | 0.53 - 1.25 | 1.83 | 0.82 - 4.11 | 2.70 # | 1.07 - 6.82 |
| From OT in recovery to NRS in recovery | 0.88 | 0.67 - 1.15 | 1.33 | 0.36 - 4.87 | 0.72 | 0.41 - 1.26 | 0.92 | 0.50 - 1.70 | 0.76 | 0.27 - 2.13 | 1.10 | 0.34 - 3.58 |
| From OT in recovery to Recovered | 0.96 | 0.68 - 1.34 | 1.10 | 0.28 - 4.33 | 0.84 | 0.46 - 1.56 | 0.68 | 0.34 - 1.35 | 0.63 | 0.20 - 1.96 | 2.75 | 0.50 - 15.28 |
| From NRS in recovery to Recovered | 0.89 ## | 0.82 - 0.96 | 1.45 | 0.87 - 2.42 | 0.80 # | 0.65 - 1.00 | 0.93 | 0.73 - 1.17 | 0.97 | 0.51 - 1.86 | 1.11 | 0.64 - 1.90 |

Notes: NRS = No Respiratory Support; OT = Oxygen Therapy; NIV = Non-Invasive Ventilation; IMV = Invasive Mechanical Ventilation; HR = Adjusted Hazard Ratio; 95% CI = 95% Confidence Interval; The multivariable model considered age, gender, SOFA score, time from COVID onset in Modena, administration of tocilizumab, glucocorticoids and lopinavir/ritonavir or darunavir/cobicistat as independent variables. ^1^ = the effect of independent variables was assessed only for changes of state that were observed in at least 10 patients; # = statistically significant at 95% confidence level (p < 0.05); ## = statistically significant at 99% confidence level (p < 0.01);
